# Metabolic effects of PCSK9 inhibition with Evolocumab in subjects with elevated Lp(a)

**DOI:** 10.1186/s12944-020-01280-0

**Published:** 2020-05-11

**Authors:** Xiang Zhang, Lotte C. A. Stiekema, Erik S. G. Stroes, Albert K. Groen

**Affiliations:** 1 Department of Experimental Vascular Medicine, Amsterdam University Medical Center, University of Amsterdam, Meibergdreef 9, 1105 AZ Amsterdam, The Netherlands; 2 Human and Animal Physiology, Wageningen University, De Elst 1, 6708 WD Wageningen, The Netherlands; 3 Department of Vascular Medicine, Amsterdam University Medical Center, University of Amsterdam, Meibergdreef 9, 1105 AZ Amsterdam, The Netherlands

**Keywords:** PCSK9 antibodies, Evolocumab, Lipoprotein(a), Metabolomics, VLDL

## Abstract

**Background:**

Epidemiological studies substantiated that subjects with elevated lipoprotein(a) [Lp(a)] have a markedly increased cardiovascular risk. Inhibition of proprotein convertase subtilisin/kexin type 9 (PCSK9) lowers both LDL cholesterol (LDL-C) as well as Lp(a), albeit modestly. Effects of PCSK9 inhibition on circulating metabolites such as lipoprotein subclasses, amino acids and fatty acids remain to be characterized.

**Methods:**

We performed nuclear magnetic resonance (NMR) metabolomics on plasma samples derived from 30 individuals with elevated Lp(a) (> 150 mg/dL). The 30 participants were randomly assigned into two groups, placebo (*N* = 14) and evolocumab (*N* = 16). We assessed the effect of 16 weeks of evolocumab 420 mg Q4W treatment on circulating metabolites by running lognormal regression analyses, and compared this to placebo. Subsequently, we assessed the interrelationship between Lp(a) and 14 lipoprotein subclasses in response to treatment with evolocumab, by running multilevel multivariate regression analyses.

**Results:**

On average, evolocumab treatment for 16 weeks resulted in a 17% (95% credible interval: 8 to 26%, *P* < 0.001) reduction of circulating Lp(a), coupled with substantial reduction of VLDL, IDL and LDL particles as well as their lipid contents. Interestingly, increasing concentrations of baseline Lp(a) were associated with larger reduction in triglyceride-rich VLDL particles after evolocumab treatment.

**Conclusions:**

Inhibition of PCSK9 with evolocumab markedly reduced VLDL particle concentrations in addition to lowering LDL-C. The extent of reduction in VLDL particles depended on the baseline level of Lp(a). Our findings suggest a marked effect of evolocumab on VLDL metabolism in subjects with elevated Lp(a).

**Trial registration:**

Clinical trial registration information is registered at ClinicalTrials.gov on April 14, 2016 with the registration number NCT02729025.

## Background

Lipoprotein(a) [Lp(a)], synthesized in the liver, is a low-density lipoprotein (LDL)-like particle covalently bound to the apolipoprotein(a) [apo(a)]. Lp(a) is cleared from the circulation primarily by the liver [1], but the exact mechanism remains to be elucidated. Epidemiological studies substantiated a markedly increased cardiovascular risk in subjects with elevated Lp(a), involving both pro-inflammatory as well as pro-coagulant effects [2]. To date, limited therapeutic options are available to reduce Lp(a) in plasma [3]. One agent that lowers Lp(a) levels is proprotein convertase subtilisin/kexin type 9 (PCSK9) monoclonal antibody, resulting in increased abundance of LDL receptors [4, 5]. The excess number of LDL receptors on the surface of hepatocytes may contribute to enhanced Lp(a) catabolism [5, 6]. However, this concept is not supported by the effect of statin therapy, which also increases abundance of the hepatic LDL receptor with a concomitant increase of plasma Lp(a) [7]. Hence, the exact role of the LDL-receptor in Lp(a) catabolism remains a matter of debate [8–10].

Besides the LDL receptor, PCSK9 also induces the degradation of the VLDL (very low density lipoprotein) receptor, ApoER2 (apolipoprotein E receptor 2), and CD36 (cluster of differentiation 36) [11, 12]. Interestingly, the VLDL receptor and CD36 are receptors for triglyceride-rich lipoproteins (TRLs) as well as Lp(a), indicating a potential interaction between Lp(a) and TRL metabolism [13–15]. A link between Lp(a) and TRL metabolism is supported by a recent NMR (nuclear magnetic resonance) metabolomics study, in which a Lp(a)-raising genotype was found to associate with decreasing concentrations of triglyceride-rich VLDL particles [16]. The mechanism underlying this inverse relationship is unknown. Another NMR metabolomics study showed that genetic inhibition of PCSK9 and statin therapy had similar effects on circulating metabolites [17]. However, genetic inhibition of PCSK9 differs from inhibition with a monoclonal antibody, since genetic inhibition includes intracellular functions of PCSK9. Moreover, data on the relationship between Lp(a) lowering and metabolism of other lipoproteins are scarce.

The aim of the current study was to investigate metabolic effects of PCSK9 inhibition (evolocumab) in patients with elevated Lp(a). To this end, we studied 30 subjects with elevated Lp(a), using either placebo (*n* = 14) or the PCSK9 inhibitor (evolocumab, *n* = 16). We analyzed NMR metabolomics of plasma derived from these participants at baseline and 16 weeks after treatment. We found that evolocumab had a marked effect on VLDL metabolism in subjects with elevated Lp(a).

## Methods

### Study design

This was a substudy of the ANITSCHKOW trial (NCT02729025) conducted at the Amsterdam UMC in The Netherlands between April 2016 and July 2017. The ANITSCHKOW trial was a phase 3b, multicenter, randomized, double-blind, placebo-controlled trial using subcutaneous injections of evolocumab 420 mg Q4W for 16 weeks as investigational product [18]. The current study was based on 30 patients (placebo *N* = 14, evolocumab *N* = 16). Written informed consent was obtained from each patient included in the study. The study protocol conforms to the ethical guidelines of the Declaration of Helsinki. The study protocol was approved by the ethic committee of the Amsterdam UMC.

The complete list of eligibility criteria for the ANITSCHKOW trial were:

- Inclusion criteria
  - Patient has provided written informed consent prior to initiation of any study specific activities/procedures
  - Male or female, ≥ 50 years of age at the time of informed consent
  - Fasting Lp(a) ≥ 125 nmol/L (50 mg/dL)
  - Fasting LDL-C ≥ 2.6 mmol/L (100 mg/dL)
  - For patients receiving lipid-lowering therapy (not required to participate in this study), lipid-lowering therapy, including statin dose, must be unchanged for ≥ 8 weeks prior to screening TBRmax above 1.6 (either right carotid, left carotid or thoracic aorta) on FDG-PET/CT
- Exclusion criteria
  - Currently receiving, or < 4 weeks since receiving, treatment in another investigational device or drug study(ies), or participating in other investigational procedures
  - Known diagnosis of diabetes mellitus or screening fasting serum glucose ≥ 7 mmol/L or glycated haemoglobin (HbA1c) ≥ 6.5%
  - History of homozygous familial hypercholesterolemia
  - Recent cardiovascular event (myocardial infarction, unstable angina, percutaneous coronary intervention [PCI], coronary artery bypass graft, or stroke) within 3 months prior to randomization, or planned cardiac surgery, PCI or carotid stenting, or planned major non-cardiac surgery during the course of the study period
  - Currently undergoing lipid apheresis
  - Known contraindications or limitations to FDG-PET/CT (eg, scanner weight limit, devices that can cause image artifacts, or carotid/aortic stents/grafts)
  - Autoimmune disease/vasculitis, active inflammatory diseases, proven or suspected bacterial infections
  - Recent (< 1 month prior to screening) or ongoing serious infection requiring intravenous antibiotic therapy
  - Recent (< 6 weeks prior to screening) or current treatment with medications that may have a significant effect on plaque inflammation as measured by plaque TBR, including: oral, rectal, or injectable corticosteroids or immunosuppressive medications (eg, cyclosporine, methotrexate, tacrolimus, azathioprine, anti-thymocyte globulin, sirolimus, anti-tumour necrosis factor agents such as infliximab, anti-interleukin [IL] 6 therapy such as tocilizumab, or anti-IL1 therapy)
  - Recent (< 6 weeks prior to screening) or current treatment with aspirin (> 325 mg/day) or nonsteroidal anti-inflammatory drugs (NSAIDs) (> 1000 mg/day)
  - Known sensitivity to any of the active substances or excipients (eg, carboxymethylcellulose) to be administered during dosing
  - Treatment with a cholesterol ester transfer protein inhibitor (eg, anacetrapib, dalcetrapib, evacetrapib) or mipomersen or lomitapide in the last 12 months prior to screening
  - Known systemic disorders such as hepatic, renal, hematologic, and malignant diseases or any clinically significant medical condition that could interfere with the conduct of the study
  - History of malignancy (except non-melanoma skin cancers, cervical in-situ carcinoma, breast ductal carcinoma in situ, or stage 1 prostate carcinoma) within the last 5 years
  - Patients likely to not be available to complete all protocol-required study visits or procedures, or unreliability as a study participant (eg, alcohol or other drug abuse in the past year or psychosis), to the best of the patient’s and investigator’s knowledge
  - History or evidence of any other clinically significant disorder, condition or disease that, in the opinion of the investigator or sponsor physician, if consulted, would pose a risk to subject safety or interfere with the study evaluation, procedures, or completion
  - Prior treatment with evolocumab or any other therapy to inhibit PCSK9
  - Pregnant or breastfeeding or planning to become pregnant or breastfeed during treatment with study drug and for an additional 15 weeks after the last dose of study drug

### Biochemical measurements

Blood samples were obtained at time of randomization and 16 weeks after treatment. Patients were fasting for ≥ 9 h for both blood withdrawals. Total cholesterol, high-density lipoprotein (HDL) cholesterol, triglycerides, and apolipoproteinB-100 (ApoB-100) were measured by commercially available kits at the Medpace core lab (Medpace Reference Laboratories, Leuven, Belgium). Low-density lipoprotein (LDL) cholesterol was calculated using the Friedewald formula. Ultracentrifugation-determined LDL-C was measured and reported if calculated LDL cholesterol was below 40 mg/dL, or triglycerides were above 400 mg/dL. Lipoprotein(a) levels were measured using an isoform-independent immunoturbidometric assay (Polymedco, Cortlandt Manor, NY, USA) and reported in nmol/L.

### Metabolite quantification

Quantification of 225 metabolic measures was performed by using a high-throughput NMR metabolomics platform (Nightingale health, Finland) [19]. The 225 metabolic measures contain around 150 primary concentrations as well as ratios that were derived from the primary concentrations. In this study, we focused on primary concentrations of circulating metabolites that cover multiple metabolic pathways including lipoproteins, fatty acids as well as amino acids and glycolysis intermediates. The following 14 lipoprotein subclasses and their lipid compositions were quantified: extremely large (average particle diameter > 75 nm), very large (average particle diameter 64.0 nm), large (53.6 nm), medium (44.5 nm), small (36.8 nm) and very small VLDL (31.3 nm); intermediate density lipoprotein (IDL; 28.6 nm); three LDL subclasses, i.e. large (25.5 nm), medium (23.0 nm) and small LDL (18.7 nm); and four HDL subclasses, i.e. very large (14.3 nm), large (12.1 nm), medium (10.9 nm) and small HDL (8.7 nm). The complete list of the 225 metabolic measures can be found at https://nightingalehealth.com/biomarkers

### Statistical analysis

#### Metabolic effects of PCSK9 inhibition with evolocumab

To assess the effect of evolocumab on levels of a circulating metabolite, we ran lognormal regression analysis. The outcome variable (*y*) was the concentration of a metabolite. Two predictor variables were in the regression model: 1) evolocumab treatment (evolocumab, *T*_*i*_ = 1; placebo *T*_*i*_ = 0); 2) metabolite concentration at time of randomization (*x*). To investigate potential combined effects of evolocumab and other lipid lowering drugs on lipoproteine subclasses, we added two indicator variables *S* and *E*. If a patient was treated with statin, then *S* = 1, otherwise *S* = 0. Similarly, if a patient was also treated with ezetimibe, then *E* = 1, otherwise *E* = 0. The baseline concentrations were centered and scaled so that the mean is 0 and standard deviation is 1. The lognormal distribution was chosen to model the outcome variable because its values were positive continuous. Due to missing observations in outcome variables, we applied the Bayesian approach to handle missing data. There were two types of missing values: (1) when the concentration of a metabolite is below the limit of detection, or (2) when values were rejected by the automatic sample and measurement quality control procedure in the Nightingale pipeline. All the missing observations were assumed missing at random and treated as parameters. Values were randomly drawn from a lognormal distribution. Regarding the missing values that were below the limit of detection, the imputed values were constrained between zero and the minimal observed value. We fitted the model by running Hamiltonian Markov Chain Monte Carlo in the program Stan (version 2.18.3). We ran four Markov chains with 2000 iterations in each chain. Results were presented with the posterior mean with 95% credible interval (CI).

#### Multilevel multivariate model

To assess the relationship between Lp(a) lowering and metabolism of 14 lipoprotein subclasses, we developed a multilevel multivariate model. The detailed model is available at https://github.com/XiangZhangSC/Anitschkow. In short, 16 subjects from the treatment group were used for this analysis, and each subject had two plasma samples at time of randomization and after treatment.

The outcome variable is a vector of 2, [*L*_*i*_, *y*_*i*_], in which *L*_*i*_ represents the Lp(a) concentration in sample *i*, and *y*_*i*_ represents the particle concentration of a lipoprotein subclass in sample *i*. The logarithm of [*L*_*i*_, *y*_*i*_] was modeled by a multivariate normal distribution, with parameters *μ*_*L*_ (a vector of 32), *μ*_*y*_ (a vector of 32) and *Σ* (a 2-by-2 covariance matrix). *μ*_*L*_[*i*] represents the mean concentration of Lp(a) in sample *i*, and was modeled as

$$ {\mu}_L\left[i\right]={\alpha}_{L, subject\left[i\right]}+{\beta}_{L, subject\left[i\right]}\times {V}_2\left[i\right] $$

*α*_*L*_ is a vector of 16, and represents the Lp(a) abundance at baseline. *β*_*L*_ is a vector of 16, and represents the effect of evolocumab treatment on Lp(a). *V*_2_ is a vector of 32 with 0 s and 1 s. When *V*_2_[*i*] = 1, it means that measurement was derived from the second visit (after treatment). Otherwise, *V*_2_[*i*] = 0. Similarly, *μ*_*y*_[*i*] represents the mean concentration of a lipoprotein subclass in sample *i*, and was modeled as

$$ {\mu}_y\left[i\right]={\alpha}_{y, subject\left[i\right]}+{\upbeta}_{y, subject\left[i\right]}\times {V}_2\left[i\right] $$

*α*_*y*_ is a vector of 16, and represents the lipoprotein subclass abundance at baseline. *β*_*y*_ is a vector of 16, and represents the effect of evolocumab treatment on the lipoprotein subclass. To assess the relationship between the Lp(a) lowering and the metabolism of a lipoprotein subclass, we used another multivariate normal distribution to model the subject-specific parameters.

$$ \left[{\alpha}_L,{\beta}_L,{\alpha}_y,{\beta}_y\right]\left[j\right]\sim MultivariateNormal\left(\left[{\theta}_L,{\gamma}_L,{\theta}_y,{\gamma}_y\right],{\varSigma}_{subj}\right) $$

*θ*_*L*_, *γ*_*L*_, *θ*_*y*_, *γ*_*y*_ and *Σ*_*subj*_ (a 4-by-4 covariance matrix) are the hyper-parameters. We fitted the model by running Hamiltonian Markov Chain Monte Carlo in the program Stan (version 2.18.3). We ran four Markov chains with 2000 iterations in each chain. Results were presented with the posterior mean with 95% credible interval (CI).

## Results

### Metabolic effects of PCSK9 inhibition with evolocumab

Baseline characteristics of the evolocumab and the placebo group were comparable (Table 1).

**Table 1:** Baseline characteristics

	Evolocumab (*n* = 16)	Placebo (*n* = 14)	P value
Age, years	58.6 (7.6)	61.4 (7.5)	0.317
Gender, n male (%)	7 (44)	7 (50)	0.732
BMI, kg/m^2^	25.5 (3.4)	26.6 (4.0)	0.493
Smoking, n active (%)	2 (13)	0 (0)	0.171
SBP, mmHg	137 (16)	139 (12)	0.574
DBP, mmHg	82 (8)	86 (8)	0.317
CVD, n (%)	3 (19)	1 (7)	0.351
Medication use, n (%)			
Statins	11 (69)	7 (50)	0.296
Ezetimibe	3 (19)	4 (29)	0.526
Total cholesterol, mmol/L^a^	5.46 (0.92)	5.62 (0.76)	0.603
LDL-cholesterol, mmol/L^a^	3.36 (0.70)	3.68 (0.68)	0.197
HDL-cholesterol, mmol/L^a^	1.46 (0.43)	1.35 (0.37)	0.519
Triglycerides, mmol/L^b^	1.38 [1.19–1.54]	1.28 [0.91–1.63]	0.533
ApoB, g/l	1.00 [0.93–1.15]	1.07 [0.92–1.17]	0.633
Lipoprotein(a), nmol/L^c^	254 [182–297]	154 [138–300]	0.430
hs-CRP, g/l	0.75 [0.58–1.58]	1.05[0.53–1.92]	0.546

Data are mean (SD), median [interquartile range], or n (%). ApoB, apolipoprotein B; BMI, body mass index; CVD, cardiovascular disease; DBP, diastolic blood pressure; HDL, high-density lipoprotein; hs-CRP, high-sensitivity C-reactive protein; LDL, low-density lipoprotein; Lp(a), lipoprotein(a); SBP, systolic blood pressure

^a^To convert to mg/dL, multiply by 38.7, ^b^ To convert to mg/dL, multiply by 88.6, ^c^ To convert to mg/dL, divide by 2.5

Wilcoxon rank-sum test was used to calculate P values for Age, BMI, SBP, DBP, Total cholesterol, LDL-cholesterol, HDL-cholesterol, Triglycerides ApoB, Lipoprotein(a) and hs-CRP

Chi square test was used to calculate *P* values for Gender, Smoking, Statins and Ezetimibe

On average, evolocumab treatment for 16 weeks resulted in a 17% (95% credible interval: [8, 26%]) reduction in Lp(a), together with a concomitant 67% [57, 76%] and 21% [6, 35%] reduction in LDL cholesterol and triglyceride, respectively.

**Fig. 1:**
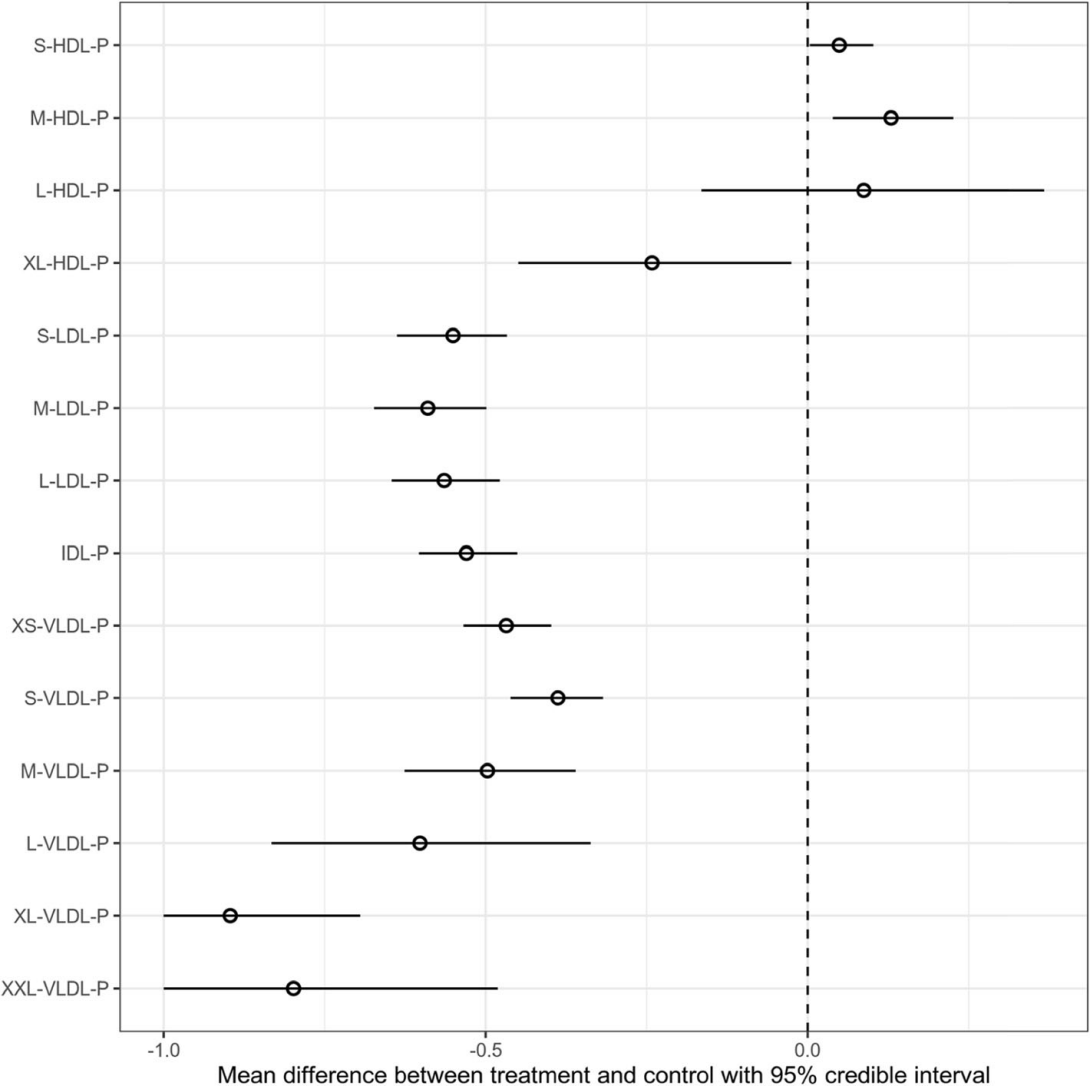
Mean difference in lipoprotein particle concentrations between evolocumab and palacebo group, adjusting for pre-treatment lipoprotein particle concentrations. Circles represent the posterior mean difference. Lines refer to the 95% credible intervals

To identify the metabolic effects corresponding to PCSK9 inhibition with evolocumab, we performed NMR metabolomics covering metabolic pathways such as lipoprotein subclasses, fatty acids, amino acids and glycolysis. We observed that evolocumab treatment resulted in substantial reduction in particle concentration of extremely large (80% [48, 100%]), very large (90% [70, 100%]), large (60% [34, 83%]), medium (50% [36, 63%]), small (39% [32, 46%]) and very small VLDL (47% [40, 53%]). We also observed that evolocumab treatment resulted in particle concentration reduction in IDL (53% [45, 60%]), large (56% [48, 65%]), medium (59% [50, 67%]) and small LDL (55% [47, 64%]). In addition, we observed that evolocumab treatment resulted in decreased concentrations of very large HDL particles (24% [3, 45%]), and increased concentrations of medium HDL particles (13% [4, 23%]) (Fig. 1).

Interestingly, we observed that evolocumab treatment had similar effects on lipoprotein subclasses in subjects with or without usage of lipid lowering medication. Compared to patients treated by statin, we observed that evolocumab treatment resulted in similar but larger effects on lipoproteins in subjects with treatment of both statin and ezetimibe (Fig. S1).

Similar to the lipoprotein particle concentration profiles, we observed that evolocumab treatment resulted in substantial reduction of esterified cholesterol (CE), triglyceride (TG), free cholesterol (FC) and phospholipid (PL) in VLDL, IDL and LDL as well as very large HDL (Fig. S2). The NMR metabolomics also quantified the fatty acid content in the lipoproteins. We observed that treatment of evolocumab resulted in 30% [24, 36%] reduction in total fatty acids, with the largest effect on docosahexaenoic acid (DHA 40% [24, 55%]) (Fig. S3). We observed no difference in concentrations of other metabolites including amino acids, fluid balance, glycolysis and ketone bodies.

### Relationship between Lp(a) lowering and reduction in lipoprotein subclasses

**Fig. 2:**
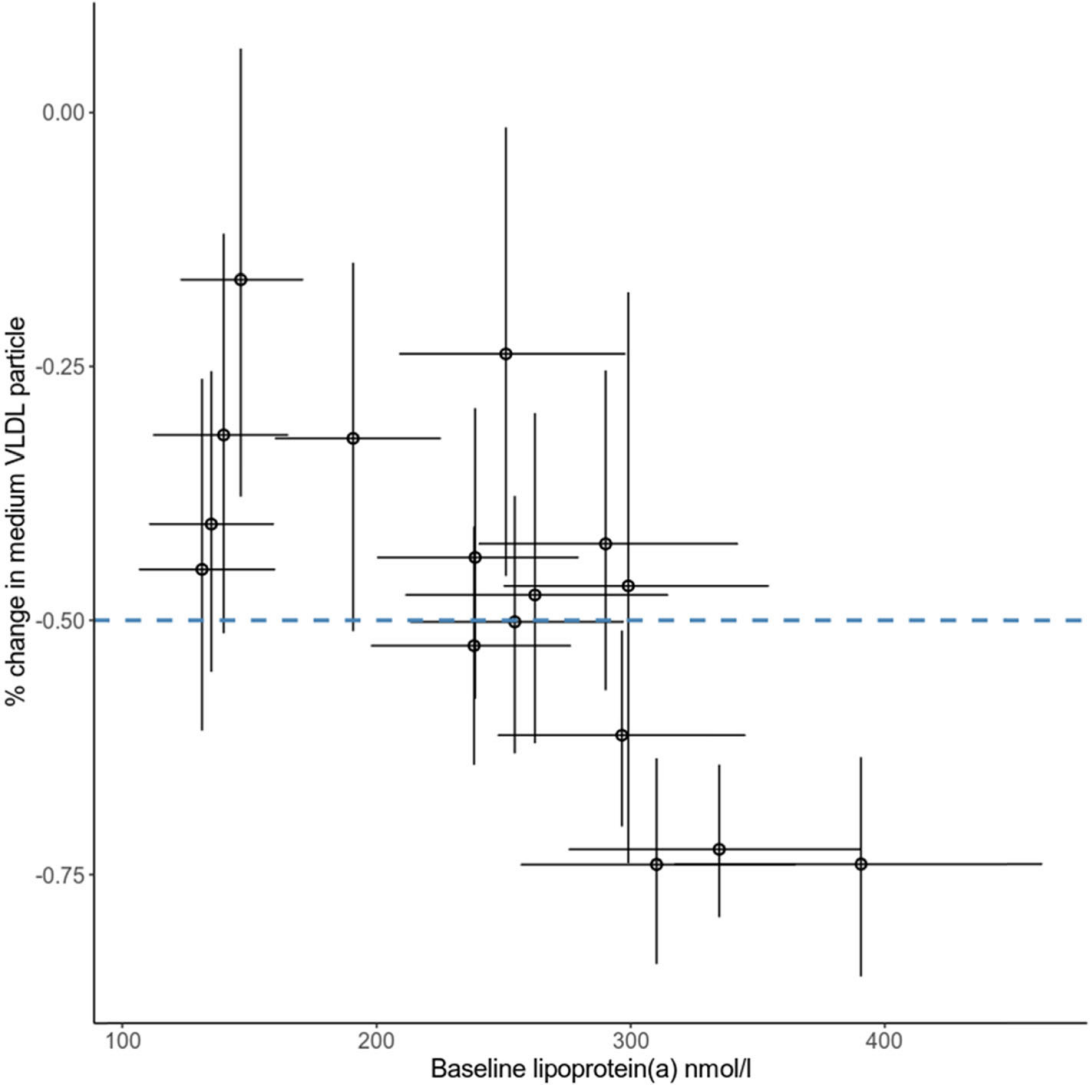
Reduction of medium VLDL particles correlated with baseline lipoprotein(a) concentrations. Every circle represents the posterior mean reduction of medium VLDL particle concentration and the posterior mean of baseline lipoprotein(a) in a patient treated with evolocumab. The vertical and horizontal bar represents the 95% credible interval. The blue dashed line represented the average percentage (50%) change in medium VLDL particle after evolocumab treatment

To identify the relationship between Lp(a) lowering and reduction in lipoprotein subclasses, we developed a multilevel multivariate model. We observed that Lp(a) lowering was not associated with reduction of the 14 lipoprotein subclasses (Fig. S4). Interestingly, we observed that the reduction in medium-sized VLDL particles was associated with increasing baseline Lp(a) concentrations (Pearson correlation coefficient − 0.5 [− 0.8, − 0.06]) (Fig. 2).

The correlations between baseline Lp(a) concentrations and reduction in other VLDL particles, including extremely large, very large, large, small and very small VLDLs, were − 0.1 [− 0.6, 0.3], − 0.03 [− 0.4, 0.4], − 0.3 [− 0.7, 0.2], − 0.1 [− 0.7, 0.6], and 0.2 [− 0.3, 0.7], respectively.

## Discussion

Here, we report that evolocumab markedly decreases VLDL particle concentrations in addition to lowering LDL-C in patients with elevated Lp(a). In particular, we identified that the extent of VLDL reduction was dependent on the baseline Lp(a) level, suggesting a marked effect of evolocumab on VLDL metabolism in subjects with elevated Lp(a).

Overall evolocumab resulted in a 17% reduction of Lp(a), a 67% reduction of LDL-C and a 21% reduction of triglycerides. Metabolomic consequences of PCSK9 inhibition with evolocumab were similar to those induced by genetic inhibition of *PCSK9* and statin therapy [17, 20]. The major metabolic effects of PCSK9 inhibition were on apoB containing lipoproteins and their lipid contents. We observed that evolocumab treatment resulted in decreasing concentration of very large HDL particles and increasing concentrations of medium sized HDL particles. Although our observation is consistent with a recent study of evolocumab on lipoprotein particles, there is no evidence suggesting that PCSK9 modulates HDL particle production or clearance [21]. Future studies are needed to investigate the role of PCSK9 in HDL metabolism. There was little effect on other circulating metabolites such as amino acids and ketone bodies. In contrast to genetic inhibition of *PCSK9* and statin therapy that had much larger effects on LDL particles than VLDL particles, we observed that evolocumab treatment induced a more substantial reduction in VLDL particles compared to LDL particles. Although we observed substantial reduction in extremely and very large VLDL particle concentrations, the reduction of triglycerides was modest. This is because the absolute concentrations of triglycerides in these two VLDL particles were very low, and the VLDLs of medium and small size were the main carriers of triglycerides (Fig. S5). With the help of detailed NMR metabolomics, we were able to visualize the changes in VLDL particles even though the overall change of triglycerides was modest. However, since this study focused on the subjects with elevated Lp(a), future studies are required to elucidate if evolocumab has similar effect on VLDL particles in subjects without elevated Lp(a). Our multivariate analysis showed that Lp(a) lowering did not correlate with particle concentration reduction in any of the 14 lipoprotein subclasses, suggesting alternative pathways for Lp(a) clearance, such as scavenger receptor class B type I (SR-BI) [22] or the plasminogen receptor [23]. Interestingly, we identified that subjects with higher baseline Lp(a) showed a larger reduction in medium VLDL particle concentrations after treatment with evolocumab. We observed that subjects with higher baseline Lp(a) also showed tendency to have a larger reduction in extremely large and large VLDL particle concentrations after treatment with evolocumab. However, those tendencies had large uncertainty due to the fact that the number of these VLDL particles present after treatment was below the detection limit, precluding assessment of correlations (Fig. S6). The number of medium VLDL particles was sufficiently high to accurately assess the relationship between its reduction and baseline Lp(a) levels. Surprisingly, we did not observe a similar relationship between baseline Lp(a) and reduction in small and very small VLDL particles, suggesting that Lp(a) may not influence catabolism of these two VLDL particles.Our observation is in line with the association between the Lp(a)-raising genotype and decreasing concentrations of extremely large, very large, large and medium VLDL particles, but not small and very small VLDL particles [16]. Lp(a) particles can bind noncovalently to triglyceride-rich lipoproteins (TRL), forming a Lp(a)-TRL complex which was suggested to facilitate receptor-mediated uptake [24, 25]. This process may be particularly important in our subjects with high Lp(a), since they may have a large amount of the Lp(a)-TRL complex. Together with the supraphysiological abundance of lipoprotein receptors induced by evolocumab treatment, these two factors may underlie the massive reduction of VLDL particles. On the other hand, apo(a) can be recycled to the extracellular space and bind to apoB-containing particles again, resulting in moderate reduction of Lp(a) [23]. Based on the above, we hypothesize that the large effect of evolocumab on VLDL particles in subjects with elevated Lp(a) is due to abundant Lp(a)-TRL complex in these patients. Future studies are required in order to test our hypothesis.

### Strengths and limitations

Our study provided the first NMR metabolomics data for a PCSK9 inhibition trial. The detailed metabolomic profiling not only allowed us to reveal systemic effects of PCSK9 inhibition, but also to assess the relationship between Lp(a) lowering and 14 lipoprotein subclasses. We did not detect statistically significant correlations between Lp(a) lowering and reduction in lipoprotein subclasses probably due to the small number of participants and considerable missing data in some lipoprotein subclasses. We feel it is worth to apply the multilevel multivariate model developed in this study in a larger clinical trial to improve our understanding of interrelationship between Lp(a) lowering and metabolism of other lipoprotein subclasses. Another limitation of this study is that we exclusively focused on the patients with elevated Lp(a) (≥ 125 nmol/L), in a future study it will be interesting to see if evolocumab treatment will result in similar effects on triglyceride-rich VLDL particles in patients with less elevated Lp(a) (for example ≥ 30 mg/dL).

## Conclusions

In conclusion, our NMR metabolomic profiling revealed that evolocumab treatment in patients with high Lp(a) markedly decreases VLDLs in addition to the well known effect on LDLs. Moreover, we found that the extent of VLDL reduction was dependent on the baseline Lp(a) level. Our findings suggest a marked effect of evolocumab on VLDL metabolism in subjects with elevated Lp(a).

## Supplementary information

**Additional file 1: Figure S1.** Mean difference in lipoprotein particle concentrations between evolocumab and palacebo group, adjusting for pre-treatment lipoprotein particle concentrations and usage of lipid lowering drugs. **Figure S2.** Mean difference in lipoprotein lipid compositions between evolocumab and placebo group, adjusting for pre-treatment lipoprotein lipid concentrations. **Figure S3.** Mean difference in fatty acid concentrations between evolocumab and placebo group, adjusting for pre-treatment fatty acid concentrations. **Figure S4.** Relationship between Lp(a) lowering and reduction in 14 lipoprotein subclasses. **Figure S5.** Triglycerides in lipoprotein subclasses. **Figure S6.** Particle concentrations of VLDLs.

## Data Availability

The datasets used and/or analysed during the current study are available from the corresponding author on reasonable request.

## Abbreviations

Lp(a): lipoprotein(a)
PCSK9: proprotein convertase subtilisin/kexin type 9
LDL-C: LDL cholesterol
NMR: nuclear magnetic resonance
VVLVLDL: extremely large VLDL
VLVLDL: very large VLDL
LVLDL: large VLDL
MVLDL: medium VLDL
SVLDL: small VLDL
VSVLDL: very small VLDL
IDL: intermediate density lipoprotein
LLDL: large LDL
MLDL: medium LDL
SLDL: small LDL
VLHDL: very large HDL
LHDL: large HDL
MHDL: medium HDL
SHDL: small HDL
