# Supplementary material for: Metabolic effects of PCSK9 inhibition with Evolocumab in subjects with elevated Lp(a)
Source: Lipids Health Dis. 2020 May 11;19:91. doi: 10.1186/s12944-020-01280-0 (PMC7216641; doi:10.1186/s12944-020-01280-0)
Supplement: Supplementary file 1 — Additional file 1: Figure S1. Mean difference in lipoprotein particle concentrations between evolocumab and palacebo group, adjusting for pre-treatment lipoprotein particle concentrations and usage of lipid lowering drugs. Figure S2. Mean difference in lipoprotein lipid compositions between evolocumab and placebo group, adjusting for pre-treatment lipoprotein lipid concentrations. Figure S3. Mean difference in fatty acid concentrations between evolocumab and placebo group, adjusting for pre-treatment fatty acid concentrations. Figure S4. Relationship between Lp(a) lowering and reduction in 14 lipoprotein subclasses. Figure S5. Triglycerides in lipoprotein subclasses. Figure S6. Particle concentrations of VLDLs. [file 12944_2020_1280_MOESM1_ESM.docx]

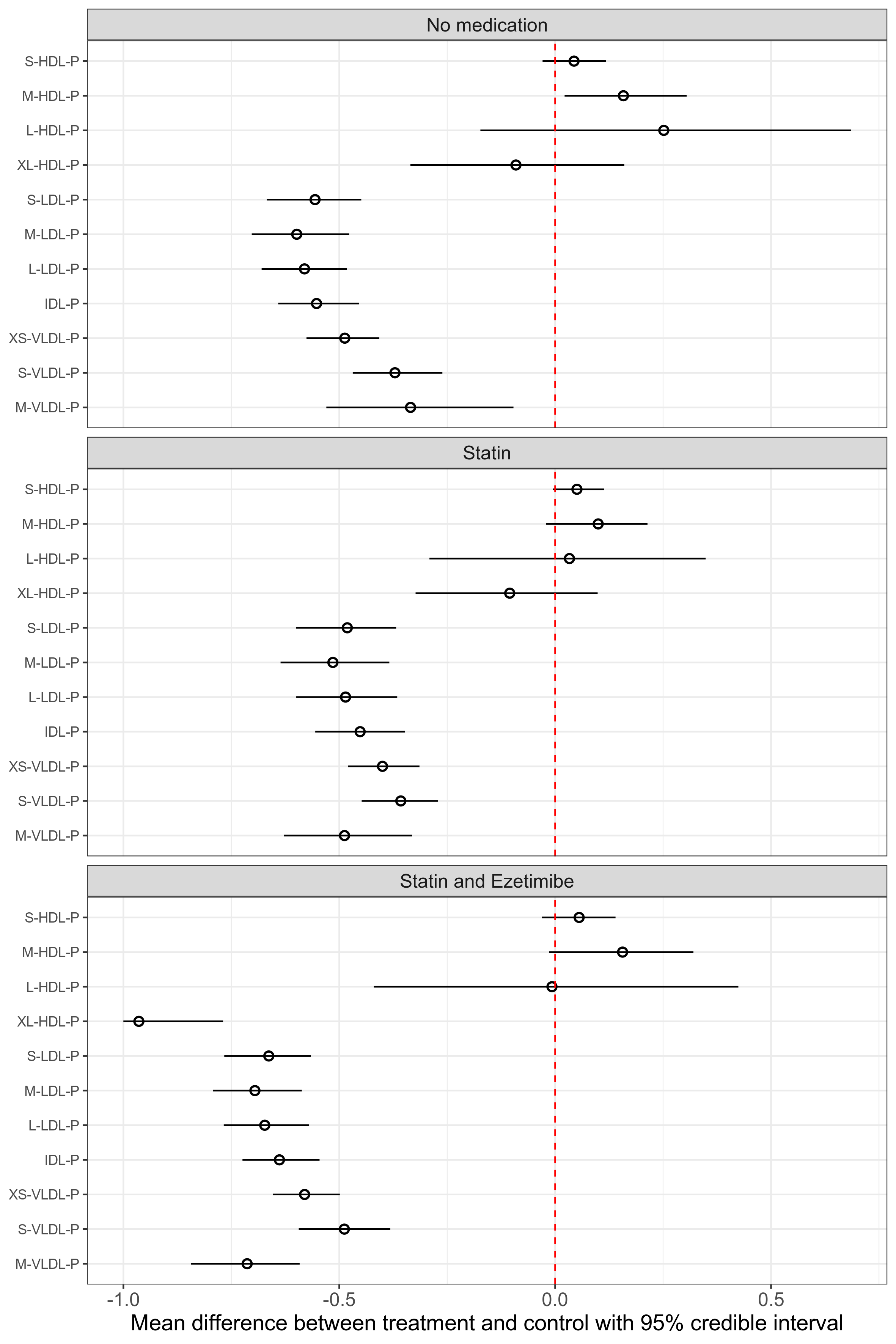


Figure S1 Mean difference between evolocumab and palacebo group, adjusting for pre-treatment lipoprotein particle concentrations and usage of lipid lowering drugs. Circles represent the posterior means. Lines refer to the 95% credible intervals


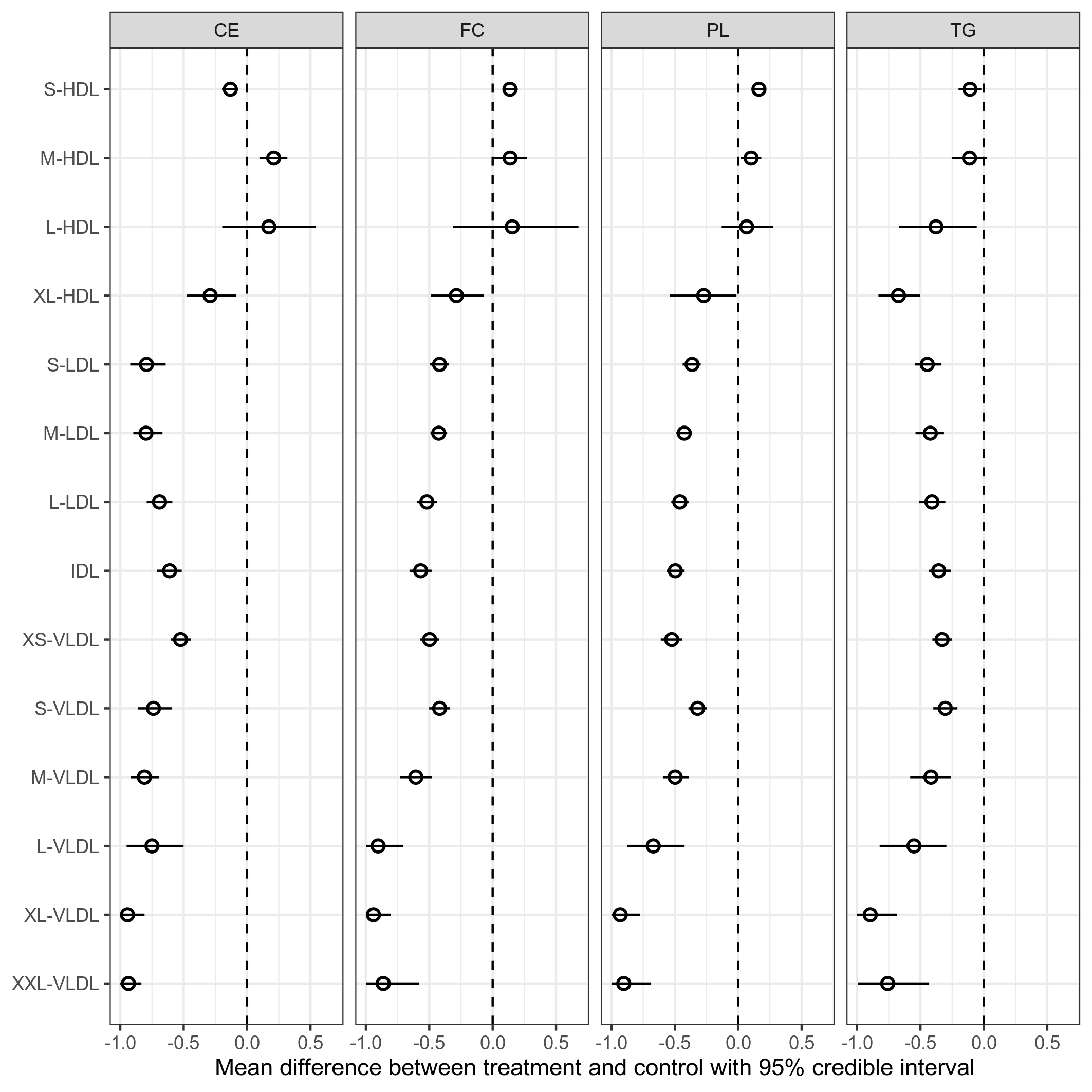


Figure S2 Mean difference in lipoprotein lipid compositions between evolocumab and placebo group, adjusting for pre-treatment lipoprotein lipid concentrations. Circles represent the posterior means. Lines refer to the 95% credible intervals. CE: esterified cholesterol; FC: free cholesterol; PL: phospholipids; TG: triglyceride.


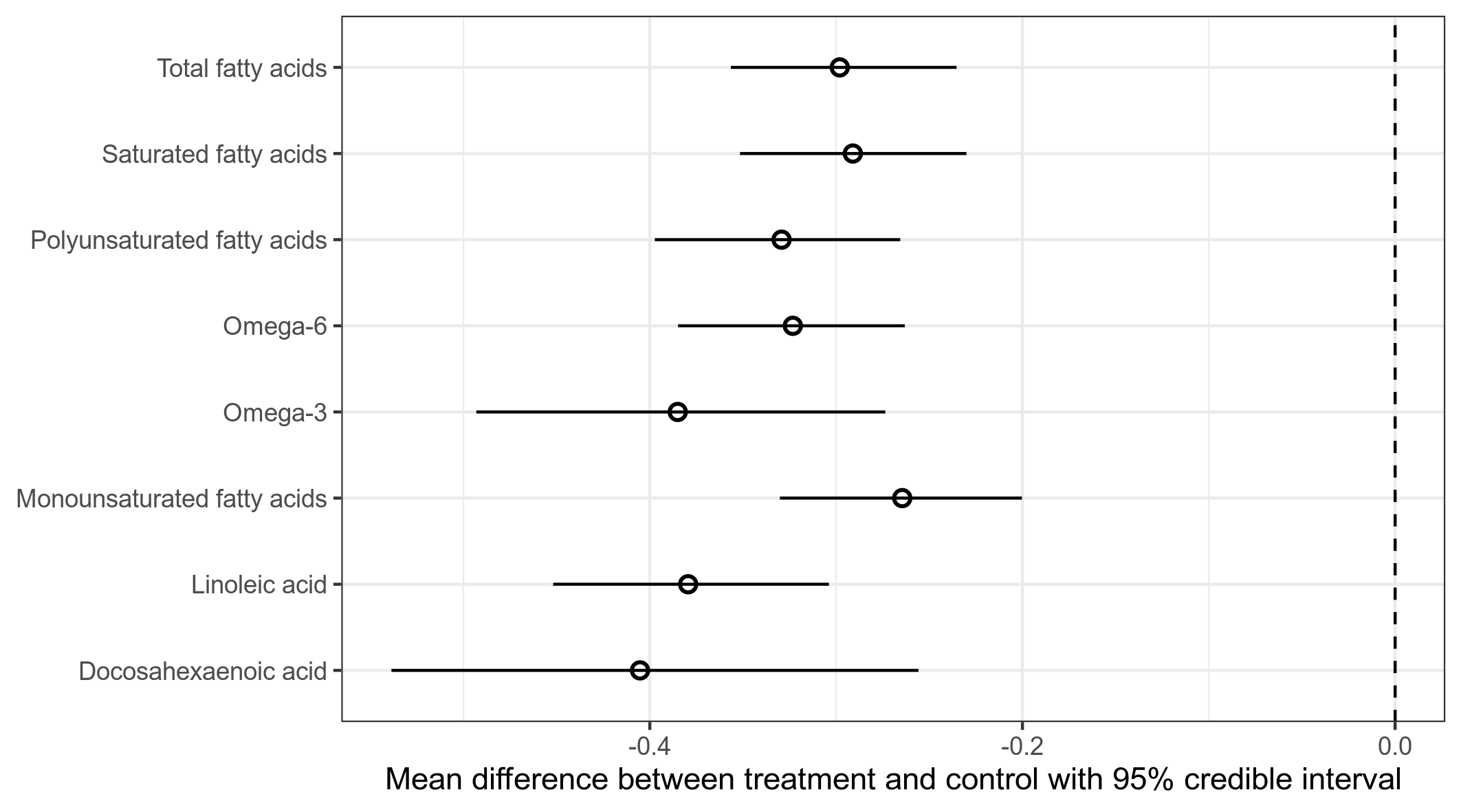


Figure S3 Mean difference in fatty acid concentrations between evolocumab and placebo group, adjusting for pre-treatment fatty acid concentrations. Circles represent the posterior means. Lines refer to the 95% credible intervals.


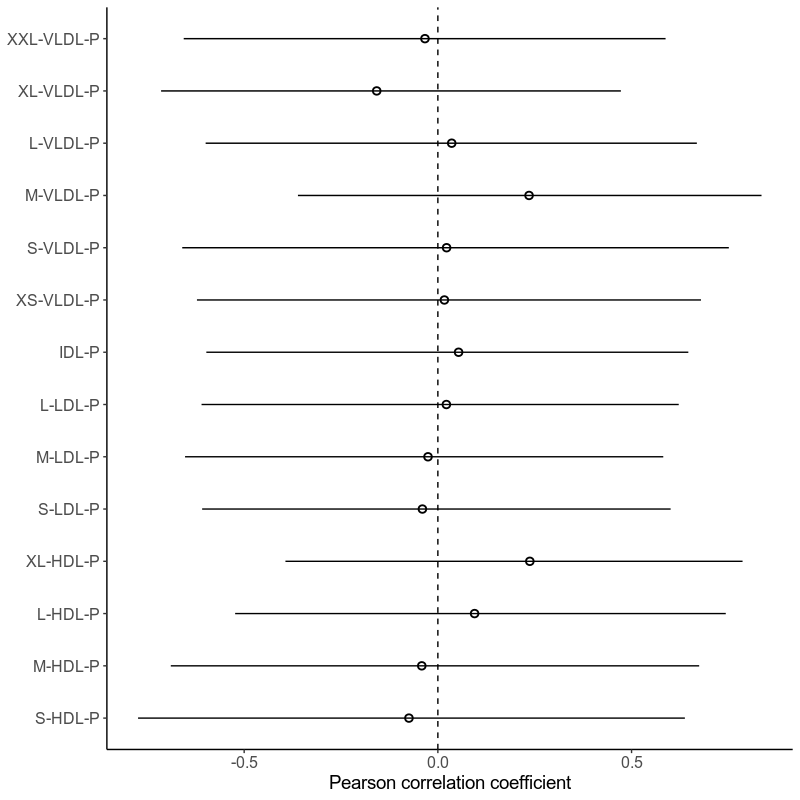


Figure S4 Relationship between Lp(a) lowering and reduction in 14 lipoprotein subclasses. Circles represent the posterior mean Pearson correlation coefficients. Lines refer to the 95% credible intervals.


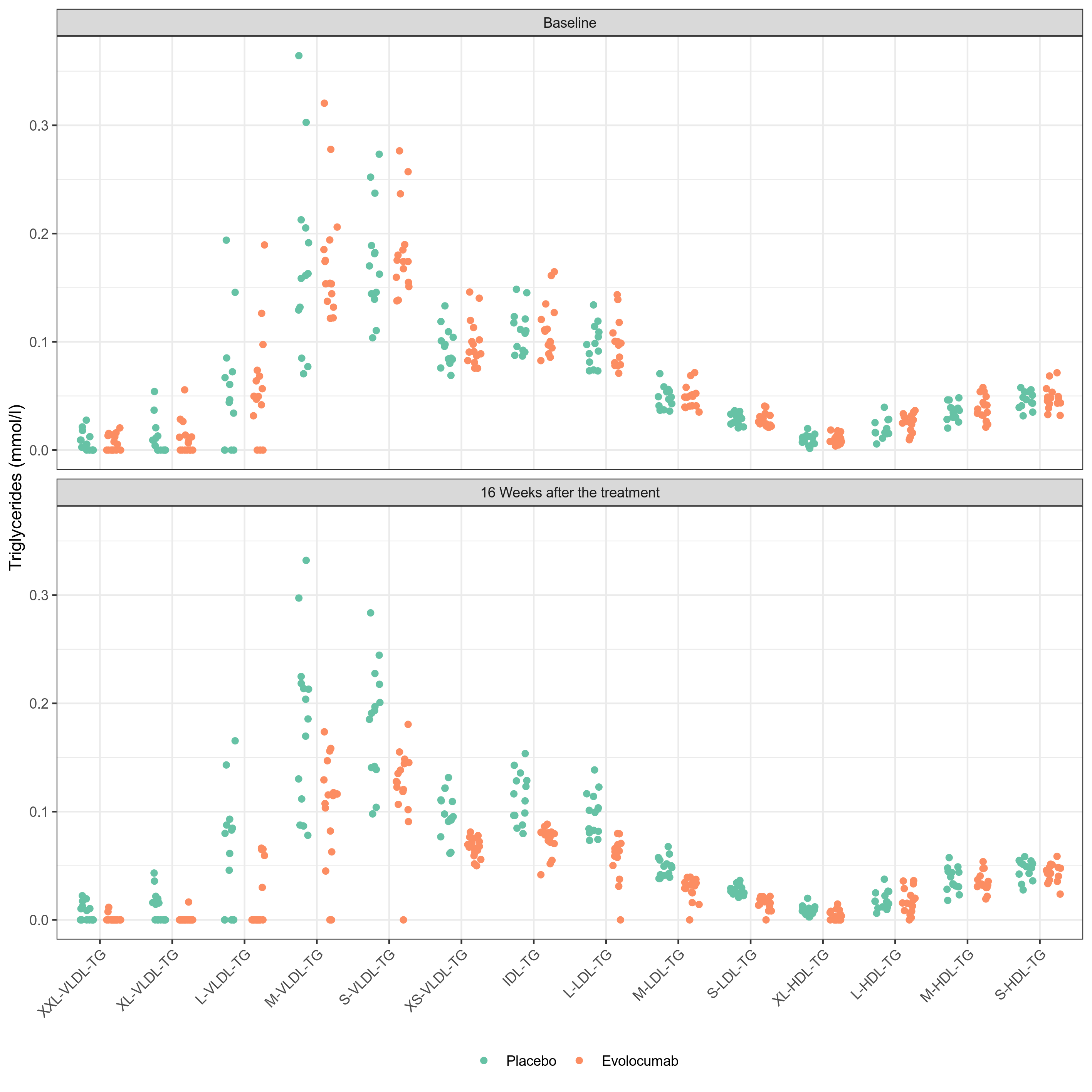


Figure S5. Triglycerides in lipoprotein subclasses


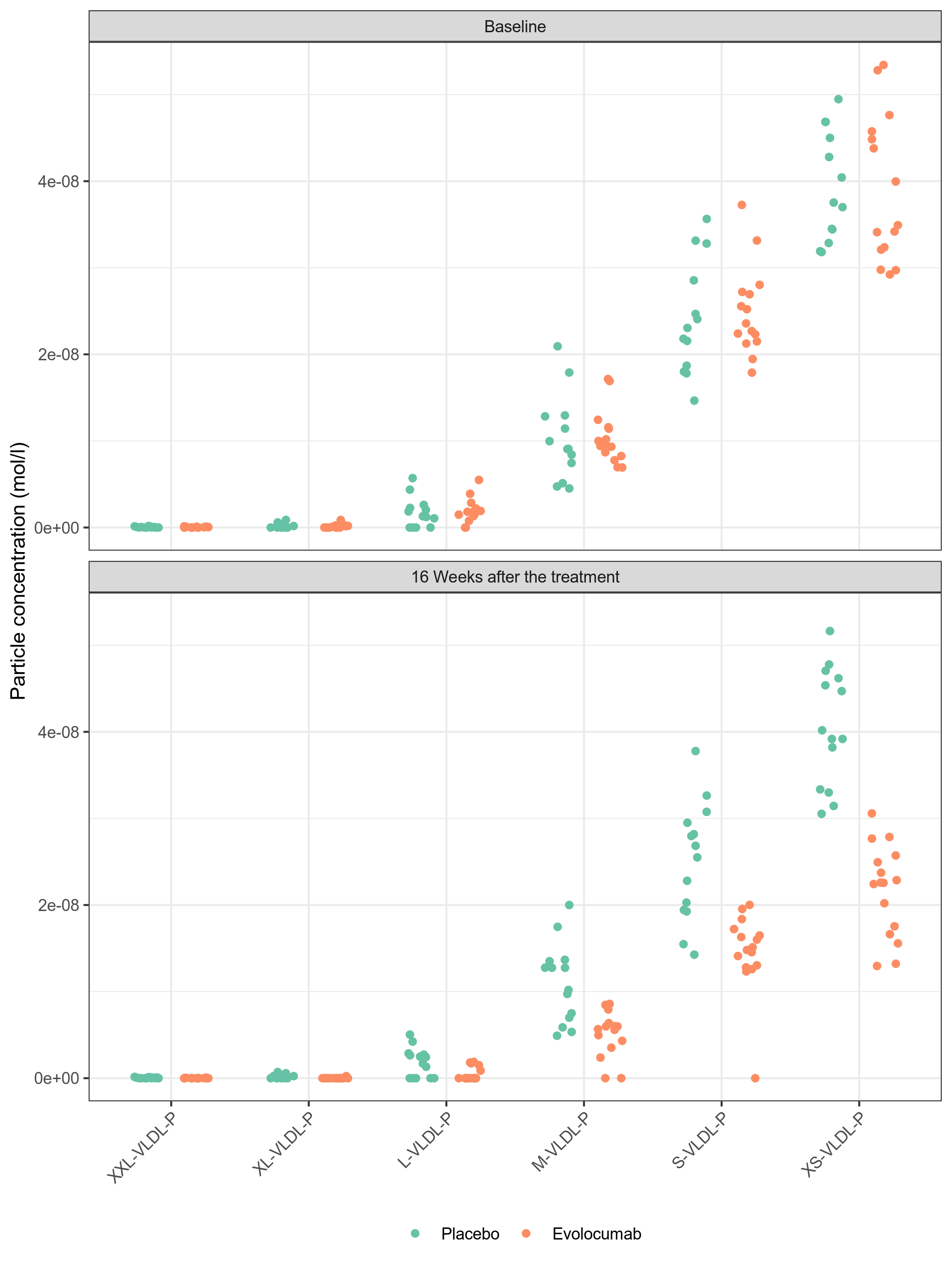


Figure S6 Particle concentrations of VLDLs.
